# Cigarette Smoke Exposure Increases Glucose-6-phosphate Dehydrogenase, Autophagy, Fibrosis, and Senescence in Kidney Cells *In Vitro* and *In Vivo*

**DOI:** 10.1155/2022/5696686

**Published:** 2022-03-27

**Authors:** Wen-Chih Liu, Hsiao-Chi Chuang, Chu-Lin Chou, Yu-Hsuan Lee, Yu-Jhe Chiu, Yung-Li Wang, Hui-Wen Chiu

**Affiliations:** ^1^Division of Nephrology, Department of Internal Medicine, Taipei Hospital, Ministry of Health and Welfare, New Taipei City, Taiwan; ^2^Department of Biology and Anatomy, National Defense Medical Center, Taipei, Taiwan; ^3^School of Respiratory Therapy, College of Medicine, Taipei Medical University, Taipei, Taiwan; ^4^Division of Pulmonary Medicine, Department of Internal Medicine, Shuang Ho Hospital, Taipei Medical University, New Taipei City, Taiwan; ^5^Division of Nephrology, Department of Internal Medicine, Shuang Ho Hospital, Taipei Medical University, New Taipei City, Taiwan; ^6^Division of Nephrology, Department of Internal Medicine, School of Medicine, College of Medicine, Taipei Medical University, Taipei, Taiwan; ^7^TMU Research Center of Urology and Kidney, Taipei Medical University, Taipei, Taiwan; ^8^Division of Nephrology, Department of Internal Medicine, Hsin Kuo Min Hospital, Taipei Medical University, Taoyuan City, Taiwan; ^9^Department of Cosmeceutics, China Medical University, Taichung, Taiwan; ^10^Graduate Institute of Clinical Medicine, College of Medicine, Taipei Medical University, Taipei, Taiwan; ^11^Department of Medical Research, Shuang Ho Hospital, Taipei Medical University, New Taipei City, Taiwan

## Abstract

Cigarette smoke (CS) is a risk factor for chronic obstructive pulmonary disease. We attempted to investigate fully the possible effects of CS on kidney cells. We found that the viability of a human kidney proximal tubular epithelial cell line (HK-2 cells) was decreased after treatment with CS extract (CSE). In particular, the effects of CSE at low concentrations did not change the expression of apoptosis and necrosis. Furthermore, CSE increased autophagy- and fibrosis-related proteins in HK-2 cells. Senescence-related proteins and the senescence-associated secretory phenotype (SASP) increased after HK-2 cells were treated with CSE. In addition, both RNA sequencing and gene set enrichment analysis data revealed that glucose-6-phosphate dehydrogenase (G6PD) in the reactive oxygen species (ROS) pathway is responsible for the changes in CSE-treated HK-2 cells. CSE increased G6PD expression and its activity. Moreover, the inhibition of G6PD activity increased senescence in HK-2 cells. The inhibition of autophagy reinforced senescence in the CSE-treated cells. In a mouse model of CS exposure, CS caused kidney damage, including tubular injury and glomerulosclerosis. CS increased fibrosis, autophagy, and G6PD expression in kidney tissue sections. In conclusion, CS induced G6PD expression, autophagy, fibrosis, and senescence in kidney cells. G6PD has a protective role in CS-induced nephrotoxicity.

## 1. Introduction

One of the main causes of death, chronic obstructive pulmonary disease (COPD), occurs in the United States [1] and worldwide [2]. Clinically, cigarette smoking (CS) is a risk factor leading to the progress of COPD [3]. Fibroblasts in the lung play a vital role in repair, regeneration, and lung homeostasis [4]. Recent studies have indicated that lung fibroblasts of patients with COPD display a decreased growth rate [5]. CS reduces the proliferation of lung fibroblasts and upregulates pathways related to cellular senescence [5] and the p53 [6], p16, and p21 retinoblastoma protein pathways [7]. Moreover, CS also induces senescence-associated secretory phenotype- (SASP-) related inflammation in human epidermal keratinocytes and skin [8]. Apoptosis resulting from smoke extract-induced COPD has been observed in *in vitro* and *in vivo* studies [9]. Furthermore, CS, nicotine, and cotinine affect red blood cell hemolysis [10]. Glucose-6-phosphate dehydrogenase (G6PD) deficiency causes substantial oxidant damage to the erythrocyte membrane [11]. A previous study reported that CS [12] and nicotine [13] increase oxidant stress to red blood cells in healthy donor volunteers. In addition, CS also induces oxidative stress as a result of reactive oxygen species (ROS) in the brain [14]. In other cases, ROS have been shown to activate transforming growth factor *β* (TGF-*β*) in the modulation of profibrotic effects [15]. ROS accumulation or antioxidant depletion occurs could destroy to cellular elements, containing DNA, RNA, proteins, lipids, and carbohydrates [16]. Early reports have shown that heat shock protein 27 (Hsp27) is a neuroprotective biomarker in ischemic stroke [17]. On the other hand, intraperitoneal injection of recombinant soluble Klotho protein improves the premature aging-related phenotype in mice with the homozygous mutated allele [18]. Klotho reduces kidney senescence and fibrosis [19] by targeting mitochondrial dysfunction in renal tubular cells [20]. CS extract (CSE) reduces the expression and secretion of Klotho in alveolar macrophages and airway epithelial cells in COPD patients [21].

CSE not only induces oxidative stress but also fibrosis-related gene expresses in orbital fibroblasts in Graves' ophthalmopathy patients [22]. Autophagy is an important and conserved “self-cleansing” pathway [23], and other studies have shown that fibrosis is often companied by autophagy [24, 25]. In the kidney, autophagy can protect the proximal tubule from damage [26] to overcome many types of kidney injury [27], aging [28], and disease [29]. Autophagy is an essential cellular process that promotes cell survival by removing protein aggregates during kidney injury [30]. However, autophagy also promotes cell death or enhances apoptosis [31]. Therefore, autophagy has two contrasting outcomes in response to stress [32]. Studies have shown that CS causes autophagy [33] and accelerates lung aging via autophagy [34]. CSE-induced autophagy regulates many cellular processes such as FOXO transcription factors in human lung adenocarcinoma cells (A549) [35] and Galectin-3 in endothelial progenitor cells [36]. In addition, SIRT1 is downregulated by autophagy in senescence and aging [37]. On the other hand, Hsp27 phosphorylation plays a crucial role in the activation of G6PD to reduce cerebral ischemia/reperfusion injury in male Wistar rats [38]. G6PD is a major source of NADPH, which drives many essential cellular processes including antioxidant pathways [39] such as the suppression of oxidative stress in cerebral ischemic male Sprague-Dawley rats [40].

Many diverse diseases may lead to chronic kidney disease (CKD) via irreversibly impaired formation or dysfunctions of the kidney, such as fibrosis [41]. Recently, a meta-analysis suggested that CS is an independent risk factor in the general adult population with CKD [42]. The database of the Korean genome and epidemiology study also revealed that the healthy middle-aged adults who smoke have a high risk of CKD [43]. COPD patients have shown renal function worsening [44]. Additionally, tobacco CS promotes kidney injuries related to biochemical changes in male adult Wistar rats [45]. In this study, we examined the effect of CS on kidney cells *in vitro* and *in vivo*. Furthermore, gene set enrichment analysis (GSEA) was performed after HK-2 cells were treated with CSE. We also observed autophagy, fibrosis, senescence, and ROS generation after CS treatment.

## 2. Material and Methods

### 2.1. Cell Line

HK-2 cells, the proximal tubular epithelial cell line from human kidney, were obtained from the American Type Culture Collection (ATCC, Manassas, VA). The cells were kept in keratinocyte-serum-free medium (K-SFM) with bovine pituitary extract (BPE) and human recombinant EGF (Invitrogen, CA), and the cells were incubated at 37°C and 5% CO_2_. The culture medium was refreshed two or three times per week. The ATG5^KD^ HK-2 cells were incubated and maintained in the K-SFM medium with rEGF and BPE at 37°C with 5% CO_2_ and cultured every two or three days. Lentivirus with control shRNA and ATG5 siRNA were purchased from the National RNAi Core Facility at Academia Sinica in Taiwan [46].

### 2.2. Preparation of Cigarette Smoke Extract

CSE solutions were prepared using a modification of standardized methods [47]. Three cigarette types (Longlife, Taipei, Taiwan; 11 mg of tar and 0.9 mg of nicotine) were subsequently collected by a liquid impinger device and then mixed with 15 ml of K-SFM. One of the cigarettes was dissolved in 5 ml of K-SFM, which was designated as 100% CSE solution.

### 2.3. Cell Viability Assay

Cell proliferation was accessed with sulforhodamine B (SRB, Sigma-Aldrich, St. Louis, MO). HK-2 cells (5 × 10^3^/well) were plated in 96-well plates and cultured with or without CSE solutions in a 37°C and 5% CO_2_ incubator overnight. After 24 h of incubation at 37°C and 5% CO_2_, the plates were moved out, washed with PBS twice, and then fixed with iced 10% trichloroacetic acid (TCA, Sigma) at 4°C for 1 h. Each well was washed 2 times with distillation-distillation H_2_O, and then, 0.1% SRB/1% acetic acid was incubated for 1 h. The wells were rinsed 2 times with 1% acetic acid and dried in an oven at 60°C for 20 min. In the end, the dye form SRB-positive cells were redissolved in 20 mM Tris buffer (Sigma) for 30 min. The absorbance was detected at a wavelength of 562 nm in an ELISA reader.

### 2.4. Flow Cytometry Analysis of Apoptosis and Necrosis

HK-2 cells were treated with CSE solutions at different doses for different times. The cells were washed with PBS and collected with Accutase (Innovative Cell Technologies, San Diego, CA). Apoptosis and necrosis were measured with a FITC Annexin V/PI apoptosis detection kit according to the manufacturer's protocol (BioLegend, San Diego, CA). The signal was detected with a flow cytometer (BD, Biosciences).

### 2.5. Lactate Dehydrogenase Assay

HK-2 cells were collected and washed in an assay medium. Cells were plated in a 96-well plate and incubated for 24 h after CSE treatment. The plate was centrifuged at 250 g for 10 min and transferred 100 *μ*l/well supernatant into corresponding wells. Cells were added to a 100 *μ*l reaction mixture and incubated for up to 30 min at room temperature. The plate was detected the absorbance at 495 nm in an ELISA reader.

### 2.6. Western Blot Analysis

All collected proteins were added to sodium dodecyl sulfate (SDS) sample buffer (62.5 mM Tris (pH 6.7), 1.25% SDS, 12.5% glycerol, and 2.5% *β*-mercaptoethanol). Proteins and the prestained protein marker (10–315 kDa) (TD-PM10315, BIOTOOLS Co., Ltd., Taipei, Taiwan) were loaded into an SDS-PAGE gel. A PVDF membrane containing transferred proteins was incubated with 5% nonfat milk with primary antibodies anti-microtubule-associated protein 1A/1B-light chain 3 (LC3) (Cell Signaling, Beverly, MA), collagen 1 (Proteintech, Rosemont IL), autophagy-related 5 (ATG5) (Proteintech), CTGF (Proteintech), PAI-1 (Cell Signaling), SIRT1 (ABclonal Inc., Woburn, MA), caspase-3 (ABclonal Inc.), Hsp27 (ABclonal Inc.), BAX (ABclonal Inc.), Klotho (Proteintech), p53 (Proteintech), p21(Proteintech), p16 (Proteintech), G6PD (Proteintech), and GAPDH (Proteintech). After the hybridization process with the abovementioned antibodies on the PVDF membrane, the membrane was rinsed with TBS-T for 15 min three times. Subsequently, the PVDF membrane was further treated with anti-mouse (Jackson) or anti-rabbit (Jackson) secondary antibody for 2 h and rinsed with TBS-T for 15 min over three times. The protein bands of the PVDF membrane were visible by performing an enhanced chemiluminescence system (Amersham, Little Chalfont, United Kingdom).

### 2.7. RNA Sequencing and Analysis

RNA sequencing used to characterize and analyze the transcriptome (RNA sequencing, Tools, Taiwan). Briefly, the purity and quantification of RNA were detected with SimpliNano™–Biochrom Spectrophotometers (Biochrom, MA, USA). The levels of RNA degradation and integrity were detected by a BiOptic Qsep100 DNA/RNA Analyzer (BiOptic Inc., Taiwan). The sequencing library was established with the KAPA mRNA HyperPrep Kit (KAPA Biosystems, Roche, Basel, Switzerland). mRNA was extracted from total RNA with magnetic oligo-dT beads and incubated at a high temperature in KAPA buffer that contained magnesium. cDNA was generated with random hexamer priming. cDNA fragments with a length of 300~400 bp were selected, and library fragments were extracted with the KAPA Pure Beads system (KAPA Biosystems, Roche, Basel, Switzerland). The library was increased with KAPA HiFi HotStart ReadyMix (KAPA Biosystems, Roche, Basel, Switzerland). Finally, the library was extracted with the KAPA Pure Beads system and qualified with the Qsep100 DNA/RNA Analyzer (BiOptic Inc). The library data were detected with high-throughput sequencing (Illumina NovaSeq 6000 platform), which was transformed into raw sequenced reads with CASAVA base calling and then stored in FASTQ format. The FASTQ files were used with FastQC and MultiQC [48]. The raw paired-end reads were filtered with Trimmomatic (v0.38) [49]. The obtained high-quality data were aligned to the reference genome (e.g., H. sapiens, GRCh38) with HISAT2 software (v2.1.0) [50, 51]. featureCounts (v1.6.0) was used to count the read numbers mapped to individual genes [52]. The RNA series dataset was uploaded to the Gene Expression Omnibus (Accession: GSE182541). GSEA was analyzed with 1000 permutations to identify enriched biological functions and activated pathways from the molecular signature database [53] (MSigDB) (https://www.gsea-msigdb.org/gsea/msigdb).

### 2.8. Quantitative Polymerase Chain Reaction (Q-PCR)

Total RNA of the kidney will be extracted using the TRIzol reagent (Invitrogen). Purity and quantification of RNA will be detected. Complementary DNA (cDNA) will be synthesized using the Easy Fast RT Kit (TOOLS, Taiwan). Q-PCR will be detected using SYBR Green (TOOLS, Taiwan). Glyceraldehyde 3-phosphate dehydrogenase (GAPDH) will be used as an internal control. The 2^−*ΔΔ*Ct^ method will be used to calculate the expression changes. All primers are listed as follows: G6PD, Hsp27, and GAPDH.

### 2.9. Senescence *β*-Galactosidase Staining

The cells were treated with CSE for 24 h, washed, fixed, and cultured in a 37°C/5% CO_2_ incubator with X-gal chromogenic substrate at pH 5.5 overnight by following the protocol for SA-*β*-gal staining (BioVision, Milpitas, CA). The images for *β*-galactosidase were collected using a digital microscope. The positive cell intensity was counted in 3 fields of view (>50 cells/field). Polydatin (MCE, Monmouth Junction, NJ) is an inhibitor of G6PD activity [54].

### 2.10. Enzyme-Linked Immunosorbent Assay

The amount of the NADPH-producing enzyme G6PD was measured using ELISA kits specific for human G6PD according to the manufacturer's protocol (Cayman Chemical, Ann Arbor, MI). The fluorescent product was measured under an excitation wavelength of 530/540 and an emission wavelength of 585-595 nm.

### 2.11. BrdU Cell Proliferation Assay

HK-2 cells were plated in a 96-well plate and incubated. BrdU was measured using BrdU Cell Proliferation Assay Kit (BioVision). Briefly, cells were added 1x 5-bromo-2-deoxyuridine (BrdU) solution and incubated plate at 37°C. Cells were fixed and denatured. Cells were hybrid with BrdU detection antibody solution. Finally, cells were added 3,3′,5,5′-tetramethylbenzidine (TMB) substrate and measured the absorbance at 650 nm.

### 2.12. Cigarette Smoke Exposure of Mouse Model

Eight-week-old male C57BL/6JNarl mice were purchased from the National Laboratory Animal Center (Taipei, Taiwan). The animal protocol was approved by the Animal and Ethics Review Committee of the Laboratory Animal Center at Taipei Medical University, Taiwan (IACUC: LAC-2017-0231). Mice were maintained under a light/dark cycle of 12 h/12 h, and the room temperature was kept at 22 ± 2°C with relative humidity of 55 ± 10%. The mouse model (*n* = 5 per group) was established by exposure to CS for 4 months. Details of the CS exposure system were reported in a previous study [55]. Briefly, the system consisted of a CS generator in a whole-body exposure chamber (TECNIPLAST, VA, Italy) with a particulate matter monitor. A side stream was placed into the whole-body exposure chamber at a flow rate of 15 l/min. Sixteen commercial cigarettes (Longlife) were combusted for 8 h/day and 5 days/week for 4 months. The mass concentration of particulate matter of <2.5 *μ*m in aerodynamic diameter was monitored using a DustTrak monitor (TSI, Shoreview, MN). The mice were sacrificed by CO_2_, and the kidneys were excised and fixed with 10% neutral formalin.

### 2.13. Histological Analysis

The kidney tissues were embedded, dehydrated, sectioned into 2 *μ*m thick slices slicing a microtome, and then stained with hematoxylin and eosin (H&E) (Sigma) for histological analysis. The glomerulosclerosis and tubular injury scores were measured. Details of the glomerulosclerosis and tubular injury scores are provided in a previously reported study [56].

### 2.14. Immunohistochemical (IHC) Staining Analysis

Kidney sections were maintained in an oven at 60°C. The kidney sections were sequentially washed with xylene (Sigma), 100% ethanol (Sigma), 95% ethanol, and 75% ethanol. Finally, the kidney sections soaked in MQ water and boiled with sodium citrate buffer (0.01 M, pH 6.0, 1% Tween 20). The sections then washed with PBS, soaked in 3% H_2_O_2_/methanol, and finally with PBS. UltraVision protein block buffer was applied to analyze the kidney after treatment with G6PD (Proteintech), LC3 (MBL) or *β*-gal (Invitrogen) antibody in 3% BSA overnight at 4°C. The sections were washed with PBS, treated with Trekkie Universal Link for 20 min, and then mixed with poly-HRP reagent for 20 min. The DAB coloring agent was used to stain the sections, followed by placement in MQ water to terminate the reaction. For the next step, hematoxylin was also used as a contrast dye for the second staining assay. In the end, the mounting buffer was added to the kidney sections, which were covered with a cover slip. Masson's trichrome staining was used according to the protocol (TRM-2-IFU, ScyTek). After the sections solidified with mounting buffer, the slices were recorded with Motic Digital Slide Assistant (Motic VM3.0, New York, NY).

### 2.15. Statistical Analysis

The results were analyzed by SPSS (SPSS Software, CA, San Diego) and plotted as the mean ± standard deviation. The statistical significance between groups was determined by Student's *t*-test. Comparisons of three or more groups were calculated by ANOVA. Significance was confirmed at *p* < 0.05.

## 3. Results

### 3.1. Cell Viability, Cell Death, and Apoptosis-Related Protein Expression in CSE-Treated HK-2 Cells

**Figure 1:**
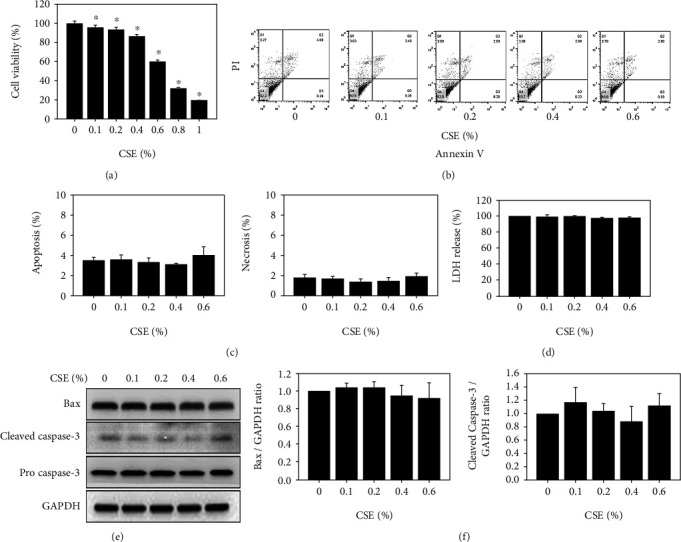
Cell viability, cell death, and apoptosis-related protein expression after treatment with CSE in HK-2 cells. (a) Cell proliferation was measured after cells were treated with CSE at concentrations of 0.1, 0.2, 0.4, 0.6, 0.8, and 1% for 24 h. ^∗^*p* < 0.05 compared to the control group. (b) The apoptosis assay of HK-2 cells was performed by flow cytometry after treatment with CSE at concentrations of 0.1, 0.2, 0.4, and 0.6% for 24 h. (c) The apoptosis and necrosis indices of HK-2 cells were measured and diagramed after treatment with CSE at concentrations of 0.1, 0.2, 0.4, and 0.6% for 24 h. (d) The LDH assay was performed after treatment with CSE at concentrations of 0.1, 0.2, 0.4, 0.6, 0.8, and 1% for 24 h in HK-2 cells. (e) The expression levels of the apoptosis-related proteins Bax and caspase 3 after treatment with CSE at concentrations of 0.1, 0.2, 0.4, and 0.6% for 24 h in HK-2 cells. (f) Bax and cleaved caspase 3 were measured and diagramed for CSE-treated cells at concentrations of 0.1, 0.2, 0.4, and 0.6% for 24 h.

The viability of CSE-treated HK-2 cells was significantly decreased in a concentration-dependent manner, as shown in Figure 1(a). After HK-2 cells were treated with CSE at low concentrations of 0.1%, 0.2%, 0.4%, and 0.6%, the cell viability decreased to 94.6%, 92.8%, 81.3%, and 67.9%, respectively. Flow cytometry analysis revealed that the CSE-treated cells at low concentrations of 0.1%, 0.2%, 0.4%, and 0.6% had no significant differences in either index of apoptosis or necrosis (Figures 1(b) and 1(c)). In addition, LDH assay revealed that the necrosis index did not have any significant differences (Figure 1(d)). Moreover, western blotting analysis showed that low concentrations of CSE did not increase Bax and cleaved caspase 3 protein expression (Figures 1(e) and 1(f)).

### 3.2. Expression of Autophagosome-Related and Fibrosis-Related Proteins in HK-2 Cells after Treatment with CSE

**Figure 2:**
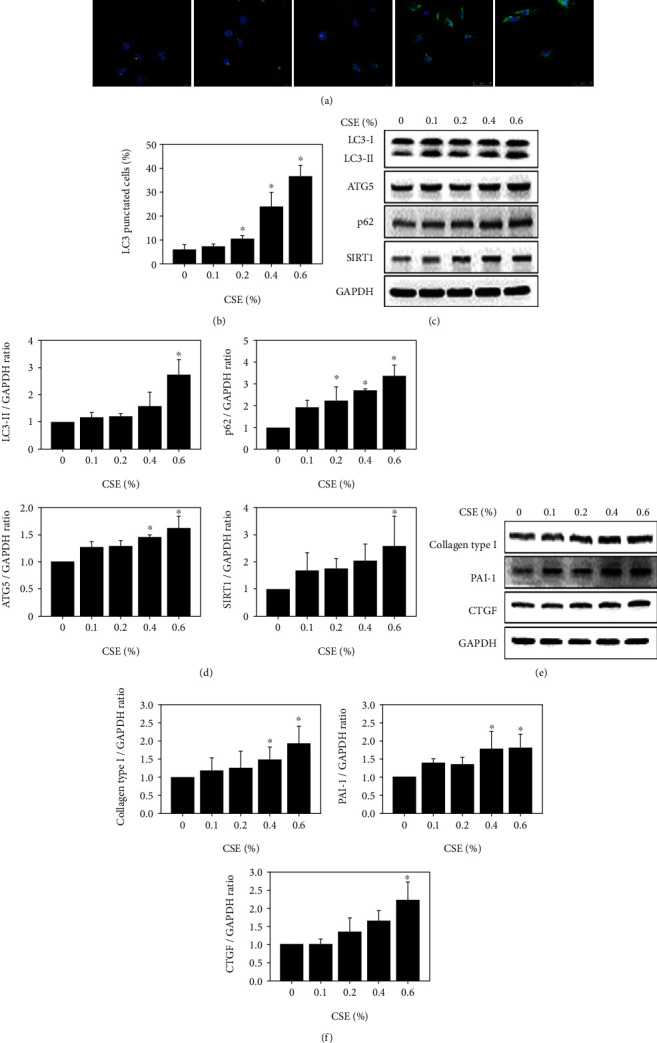
Autophagosome-related protein, SIRT1, and fibrosis-related protein expression after HK-2 cells were treated with CSE. (a) The expression levels of LC3 (green color) were measured in HK-2 cells after treatment with CSE at concentrations of 0.1, 0.2, 0.4, and 0.6% for 24 h. 4′,6-Diamidino-2-phenylindole (DAPI) (blue color) was used to stain nuclei. Scale bar: 50 *μ*m. (b) Images of LC3-punctuated cells treated with CSE were graphed and statistically analyzed at concentrations of 0.1, 0.2, 0.4, and 0.6% for 24 h. ^∗^*p* < 0.05 compared to the control group. (c) Western blot showing the expression of SIRT1 and the autophagy-related proteins p62, ATG5, and LC3 after CSE treatment at concentrations of 0.1, 0.2, 0.4, and 0.6% for 24 h. (d) Protein expression of LC3-II, p62, ATG5, and SIRT1 was measured and diagramed for CSE-treated cells at concentrations of 0.1, 0.2, 0.4, and 0.6% for 24 h. ^∗^*p* < 0.05 compared to the control group. (e) The expression levels of the fibrosis-related proteins collagen type 1, PAI-1, and CTGF after treatment with CSE at concentrations of 0.1, 0.2, 0.4, and 0.6% for 24 h. (f) Collagen type 1, PAI-1, and CTGF were measured and diagramed in HK-2 cells after treatment with CSE at concentrations of 0.1, 0.2, 0.4, and 0.6% for 24 h. ^∗^*p* < 0.05 compared to the control group.

The expression levels of LC3 determined by immunofluorescence were concentration-dependent (Figure 2(a)). Statistical analysis also showed that LC3 levels were CSE concentration-dependent in HK-2 cells (Figure 2(b)). Approximately 35 ± 2.5% of HK-2 cells expressed LC3 signals after treatment with 0.6% CSE. Furthermore, CSE-treated HK-2 cells revealed higher expression of the autophagy-related proteins LC3, p62, ATG5, and SIRT1, as determined by western blotting (Figure 2(c)). The expression levels of LC3, p62, ATG5, and SIRT1 were also concentration-dependent in HK-2 cells after CSE treatment (Figure 2(d)). On the other hand, HK-2 cells exposed to CSE exhibited higher expression of the fibrosis-related proteins collagen type 1, PAI-1, and CTGF, as determined by western blotting (Figure 2(e)). Statistical analysis also revealed that the levels of collagen type 1, PAI-1, and CTGF after treatment with CSE in HK-2 cells were also concentration-dependent (Figure 2(f)).

### 3.3. Senescence, Senescence-Related Proteins, and Senescence-Associated Secretory Phenotype-Related Inflammation in HK-2 Cells after CSE Treatment

**Figure 3:**
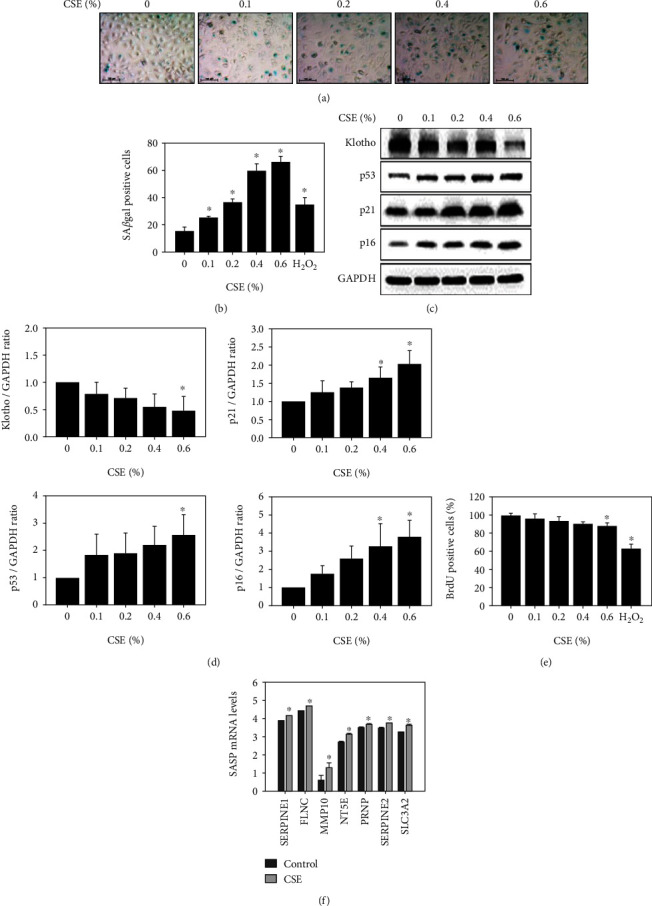
Senescence, senescence-related proteins, and senescence-associated secretory phenotype-related inflammation in HK-2 cells after treatment with CSE. (a) The expression of SA*β*gal (turquoise color) was detected after treatment with CSE at concentrations of 0.1, 0.2, 0.4, and 0.6% for 24 h. (b) The results of SA*β*gal-positive cells were graphed and statistically analyzed after treatment with CSE at concentrations of 0.1, 0.2, 0.4, and 0.6% for 24 h. ^∗^*p* < 0.05 compared to the control group. (c) The expression of senescence-related proteins Klotho, p53, p21, and p16 in CSE-treated cells at concentrations of 0.1, 0.2, 0.4, and 0.6% for 24 h. (d) Klotho, p53, p21, and p16 were graphed and analyzed after cells were treated with CSE at concentrations of 0.1, 0.2, 0.4, and 0.6% for 24 h. ^∗^*p* < 0.05 compared to the control group. (e) The results of BrdU-positive cells were graphed and statistically analyzed after treatment with CSE at concentrations of 0.1, 0.2, 0.4, and 0.6% for 24 h. ^∗^*p* < 0.05 compared to the control group. H_2_O_2_ served as a positive control. (f) SASP was graphed and statistically analyzed from RNA sequencing data of CSE-treated HK-2 cells following 24 h. ^∗^*p* < 0.05 compared to the control group.

Senescence-positive cells were observed in concentration-dependent manner as determined by the SA*β*gal assay as shown in Figure 3(a). Analysis of SA*β*gal-positive CSE-treated HK-2 cells also demonstrated concentration dependence (Figure 3(b)). CSE-treated HK-2 cells displayed higher expression of the senescence-related proteins p53, p21, and p16 as determined by western blotting (Figure 3(c)). A previous study showed that Klotho reduced kidney senescence and fibrosis [19]. Klotho exhibited lower expression after treatment with CSE (Figure 3(c)). p53, p21, p16, and Klotho levels in HK-2 cells treated with CSE were also concentration-dependent (Figure 3(d)). Senescent cells have inhibited cellular proliferation [57], which can be detected by BrdU. BrdU-positive HK-2 cells treated with CSE were also observed to be concentration-dependent (Figure 3(e)). Furthermore, SLC3A2, SERPINE2, PRNP, NT5E, MMP10, FLNC, and SERPINE1 showed higher expression after treatment with CSE in HK-2 cells, as determined by RNA sequencing (Figure 3(f)).

### 3.4. RNA Sequencing, Gene Set Enrichment Analysis, and Interpretative Phenomenological Analysis in CSE-Treated HK-2 Cells

**Figure 4:**
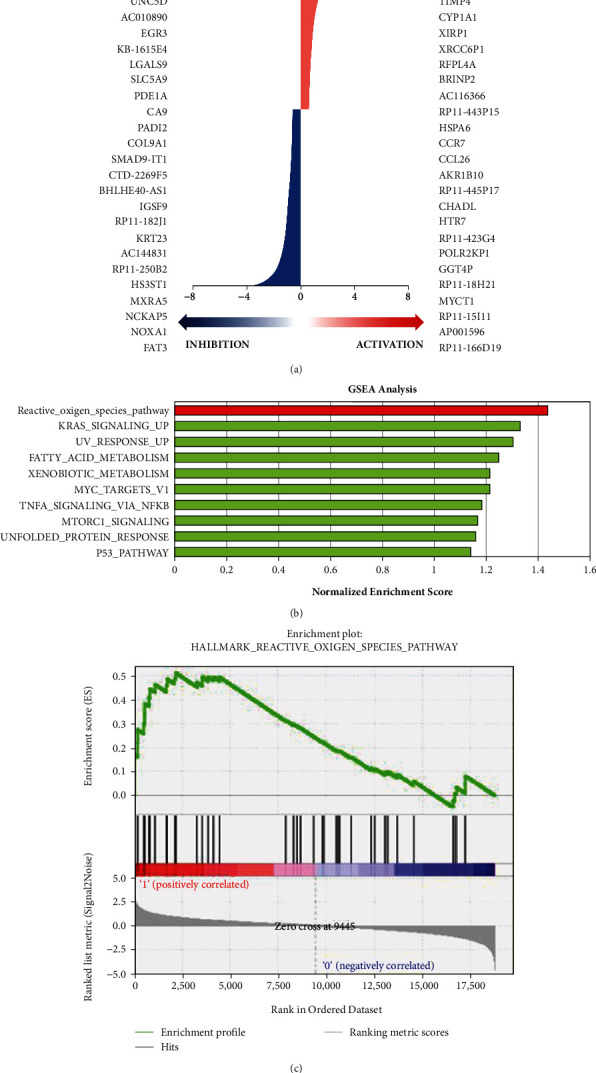
RNA sequencing and gene set enrichment analysis of CSE-treated HK-2 cells. (a) RNA was extracted from HK-2 cells and sequenced after treatment with or without CSE at a concentration of 0.6%. Upregulation and downregulation of mRNA are presented as the fold change −0.58 < FC > 0.58. (b) GSEA was used to analyze the pathways of HK-2 cells after treatment with CSE and showed a normalized enrichment score. (c) The Hallmark reactive oxygen species gene set database of the enrichment plot was used as the gene set collection for analysis.

The RNA sequencing data after treatment of HK-2 cells with CSE are shown in Figure 4(a). The most upregulated gene was MMP3 in CSE-treated cells, while the most downregulated gene was SEMA5B. In addition, the data were further analyzed by GSEA (Figure 4(b)). The results showed higher expression of the ROS pathway in HK-2 cells after treatment with CSE (Figure 4(b)). The enrichment plot showed higher enrichment of the ROS pathway after cells were exposed to CSE (Figure 4(c)). Moreover, ROS-related gene expression is shown in Table 1. The most upregulated gene was G6PD in HK-2 cells after CSE treatment as shown in Table 1.

### 3.5. Expression Profile of Heat Shock Protein 27, Glucose-6-phosphate Dehydrogenase, and Senescence-Associated *β*-Galactosidase Assay Regulation with Autophagy in CSE-Treated HK-2 Cells

**Figure 5:**
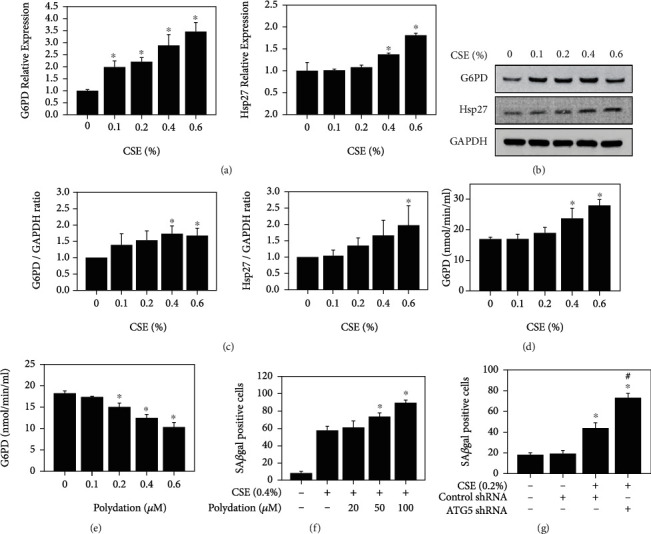
The expression profile of heat shock protein 27 and glucose-6-phosphate dehydrogenase, activity of glucose-6-phosphate dehydrogenase, and senescence-associated *β*-galactosidase assay with polydatin or ATG shRNA in HK-2 cells after treatment with CSE. (a) Relative expression in Hsp27 and G6PD mRNA was measured in CSE-treated cells at concentrations of 0.1, 0.2, 0.4, and 0.6% for 24 h. ^∗^*p* < 0.05 compared to the control group. (b) The expression of Hsp27 and G6PD in HK-2 cells after treatment with CSE at concentrations of 0.1, 0.2, 0.4, and 0.6% for 24 h. ^∗^*p* < 0.05 compared to the control group. (c) Hsp27 and G6PD expressions were graphed and analyzed for CSE-treated cells at concentrations of 0.1, 0.2, 0.4, and 0.6% for 24 h. ^∗^*p* < 0.05 compared to the control group. (d) The activation of G6PD was detected after CSE treatment at concentrations of 0.1, 0.2, 0.4, and 0.6% for 24 h. ^∗^*p* < 0.05 compared to the control group. (e) The expression of G6PD activation was detected after polydatin treatment at concentrations of 10, 20, 50, and 100 *μ*M for 24 h. ^∗^*p* < 0.05 compared to the control group. (f) SA*β*gal expression was detected in cells after treatment with CSE at concentrations of 0.6% and polydatin at concentrations of 10, 20, 50, and 100 *μ*M for 24 h. ∗p <0.05, CSE compared to CSE+polydatin. (g) The expression of SA*β*gal was measured after treatment with CSE, control shRNA, and ATG5 shRNA at concentrations of 100 *μ*M for 24 h. ^∗^*p* < 0.05 compared to the control group. ^#^*p* < 0.05, CSE+control shRNA compared to CSE+ATG5 shRNA.

Hsp27 and G6PD were observed to be concentration-dependently increased, as determined by real-time polymerase chain reaction (Q-PCR) assay (Figure 5(a)) and western blotting (Figure 5(b)). The expression of Hsp27 and G6PD in HK-2 cells after treatment with CSE was also concentration-dependent (Figure 5(c)). The G6PD activity after HK-2 cell treatment with CSE was also concentration-dependent (Figure 5(d)). G6PD activity was higher in HK-2 cells after CSE treatment at concentrations of 0.6% and decreased after polydatin treatment at concentrations of 10, 20, 50, and 100 *μ*M (Figure 5(e)). Furthermore, the statistical expression of SA*β*gal-positive cells was increased in CSE-treated HK-2 cells, and the expression increased at concentrations of 20, 50, and 100 *μ*M after treatment with polydatin (Figure 5(f)). In addition, the number of SA*β*gal-positive cells treated with both ATG5 shRNA and control shRNA was increased after CSE exposure compared with that in the control groups (Figure 5(g)). Moreover, ATG5 shRNA enhanced the number of CSE-induced SA*β*gal-positive cells.

### 3.6. Tubular Injury and Glomerulosclerosis Score Analysis of Senescence, Senescence-Associated *β*-Galactosidase, Autophagy Protein LC3, and Glucose-6-phosphate Dehydrogenase after treatment of Mice with CS

**Figure 6:**
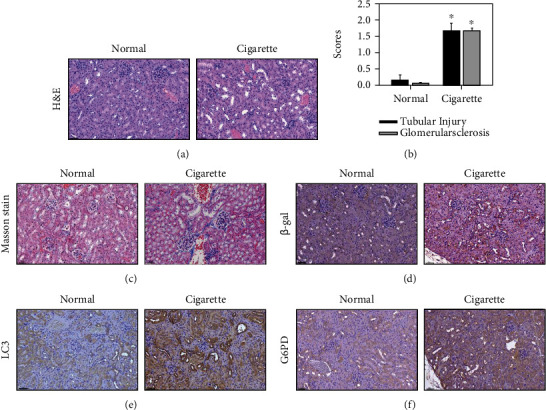
Hematoxylin and eosin staining in kidney samples. Tubular injury, glomerulosclerosis score, and Masson staining after treatment of mice with CS. (a) C57BL/6 mice were treated with CSE and harvested for 4 months. H&E staining was examined in kidney samples. The cell nuclei were stained blue by hematoxylin. Both the extracellular matrix and cytoplasm were stained by eosin (pink). Scale bar: 50 *μ*m. (b) Tubular injury and glomerulosclerosis were analyzed in kidneys (*N* = 5). The data are presented as the means ± SD. Twenty fields of view per kidney. ^∗^*p* < 0.05 and ^∗∗∗^*p* < 0.001 compared to the normal group samples. (c) Kidneys were stained with Masson's trichrome. Scale bar = 50 *μ*m. Immunohistochemistry for senescence-associated *β*-galactosidase, autophagy-related proteins, glucose-6-phosphate dehydrogenase, and kidney samples of after treatment of mice with CSE. (d) C57BL/6 mice were treated with CSE and then harvested after 4 months. IHC staining of SA*β*gal was examined in kidney samples. (e) IHC staining of LC3 was examined in kidney samples. (f) IHC staining of G6PD was examined in kidney samples. Scale bar: 50 *μ*m.

A mouse model was established by exposure of mice to CS for 4 months. H&E staining displayed varying degrees of tubular injury and glomerulosclerosis when comparing the CS-treated group to the normal group (Figure 6(a)). The tubular injury and glomerulosclerosis scores were significantly increased after treatment of mice with CS (Figure 6(b)). The kidney sample also revealed varying degrees of fibrosis staining with Masson's trichrome (purple) (Figure 6(c)). IHC staining showed higher expression of SA*β*gal, LC3 and G6PD than that of the normal group at 4 months (Figures 6(d)–6(f)).

## 4. Discussion

A previous study showed that CS altered cell viability in gingival mesenchymal cells at a concentration of 250 *μ*g/ml [58]. The cell viability of mouse embryonic fibroblasts and NIH3T3 cells decreased below 50% after exposure to 400 *μ*l of 4% CSE solution [59]. In addition, the cell viability of human lung bronchial epithelial cells (BEAS-2B) decreased to 50% after treatment with 10-20% CSE [60]. Our results showed that cell viability decreased by over 50% in HK-2 kidney cells after treatment with CSE (0.8% and 1%) (Figure 1(a)). Early reports indicated that apoptosis was significantly induced in BEAS-2B cells by CSE [61]. However, our results showed that low concentrations CSE (0.1%-0.6%) did not induce apoptosis in HK-2 cells (Figures 1(b)–1(e)). A previous study showed that the CSE-induced autophagy in A549 cells is associated with many cellular processes [35]. Our results indicated that CSE-treated HK-2 cells not only induced autophagy but also induced SIRT1 after CSE (0-0.6%) exposure (Figures 2(a)–2(d)). Hence, our results indicated that autophagy was regulated by SIRT1, which has been reported in other studies [62–64].

CSE is a risk factor for the development of lung fibrosis [65]. Renal fibrosis is involved in various kidney diseases [66]. Previous studies have demonstrated that through the autocrine and paracrine stimulation of cells by TGF-*β*1, CTGF is released and synthesized, which plays a role in fibrogenesis [67]. Furthermore, PAI-1 is the major physiologic inhibitor of the plasmin-based pericellular cascade and a causative factor in the fibroproliferative disorders [68]. The upregulation of CTGF and PAI-1 caused extracellular matrix (ECM) accumulation [69, 70]. In the current study, CSE exhibited higher expression of the fibrosis-related proteins including collagen type 1, PAI-1, and CTGF in HK-2 cells (Figures 2(e) and 2(f)). In *in vivo* study, the significant accumulation of collagen fibers in the kidney tissues of the CS group (Figure 6(c)). These results indicated that CS may cause kidney fibrosis. Previous research has shown that CS induces SASP-related inflammation in human epidermal keratinocytes and skin [8]. The expression of fibrosis-related genes is induced in orbital fibroblasts from patients with Graves' ophthalmopathy [22]. In addition, previous study revealed that CS reduced the proliferation of lung fibroblasts by upregulating signaling pathways such as cell senescence, p53, and p16-retinoblastoma [5]. The p53 [6], p16, and p21 pathways [7] were related to cellular senescence, including fibrosis accompanied by senescence, senescence-related proteins, and SASP-related inflammation (Figures 3(a)–3(f) and Figure 6(d)). Early reports showed that CSE reduces the expression and secretion of Klotho in alveolar macrophages and airway epithelial cells in COPD patients [21]. The present study demonstrated that Klotho significantly decreased in human kidney cells after the CSE (0%-0.6%) treatment (Figure 3(c)).

Recently, a meta-analysis suggested that CS is an independent risk factor for the general adult population with CKD [42] and healthy middle-aged adults [43]. In addition, GSEA revealed that three pathways were involved in CS-treated BEAS-2B cells, namely, cell matrix adhesion and the TGF-*β* receptor signaling pathway, RNA catabolic processes, and the regulation of cell cycle phase transition, as well as calcium-mediated signaling and regulation of cell-cell adhesion [71]. Deficiency of G6PD in the erythrocyte membrane causes substantial oxidant damage [11]. G6PD is a major source of NADPH that is involved in antioxidant pathways [39]. Furthermore, GSEA showed that the ROS pathway is the primary regulator in HK-2 cells after treatment with CSE. In particular, the expression of G6PD increased in the ROS-related gene expression pathway after treatment with CSE (Figures 4(a)–4(c) and Table 1). G6PD and Hsp27 were highly expressed in CSE-treated HK-2 cells (Figures 5(a)–5(c)). On the other hand, Hsp27 phosphorylation plays an important role in the activation of G6PD [38], and the phosphorylation of G6PD results in a reduction of NADPH, subsequently causing oxidative stress which may lead to metabolic syndromes [72, 73]. Polydatin is a new inhibitor of G6PD that can block the pentose phosphate pathway [54]. Our results showed that polydatin decreased the activity of G6PD in a dose-dependent manner (Figure 5(e) and Table 1). The expression of senescence-related factors was increased after treatment with polydatin in HK-2 cells (Figure 5(f)). These data showed that G6PD plays an important role in the protection of kidney cells. A previous study indicated that CS accelerated lung aging [34] and kidney injury [74] via autophagy. Our results showed that CSE induced autophagy (Figures 2(a)–2(d)). Here, we used ATG5 shRNA to increase senescence expression in HK-2 cells (Figure 5(g)) and found that CSE-induced autophagy may inhibit senescence and has a protective role in kidney cells.

## 5. Conclusions

**Figure 7:**
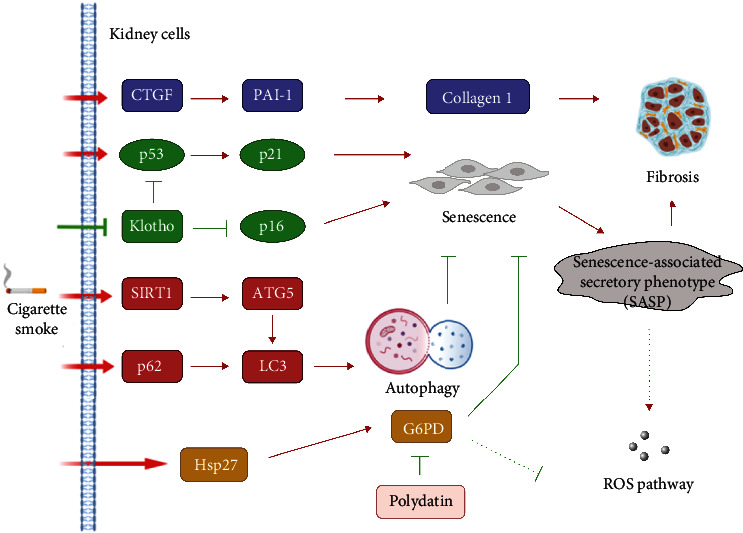
Schematic of the putative mechanism illustrating the CSE-induced G6PD, autophagy-related, fibrosis-related, and senescence-related protein expressions in kidney cells. Kidney cells were induced by CSE via several related pathways that increase the expression of the fibrosis-related proteins, autophagy-related proteins, and senescence-related proteins in kidney cells. CSE regulates senescence and fibrosis via inhibition of Hsp27, G6PD, Klotho, and autophagy.

We found that CSE induced autophagy, fibrosis, senescence, and SASP in kidney cells (Figure 7). In contrast, Klotho expression was decreased in kidney cells after CSE treatment. Furthermore, RNA sequencing and GSEA revealed that G6PD played an important role in ROS pathway regulation in kidney cells after CSE exposure. G6PD expression and G6PD activity increased in CSE-treated kidney cells. In addition, G6PD inhibited senescence in kidney cells. In an animal model after CS exposure for 4 months, CS caused tubular injury and glomerulosclerosis and induced fibrosis, autophagy, and G6PD. In conclusion, CS induced G6PD, autophagy, fibrosis, and senescence and decreased Klotho in kidney cells. These findings offer more precise molecular mechanism of CS and the chance to find potential preventive or therapeutic strategies for CS-related renal injury. In the current study, we focus on *in vitro* study and an animal model. In the future, we hope to utilize clinical data and samples for validation of the research that we currently perform on in vitro and animal models.

## Figures and Tables

**Table 1:** The top 20 genes of Hallmark reactive oxygen species gene set.

Gene	FC	*p* value
G6PD	0.979605	6.56*E* − 76
GCLM	0.960879	3.98*E* − 50
SOD1	0.952822	2.29*E* − 67
TXN	0.843715	4.23*E* − 36
HHEX	0.820415	3.26*E* − 08
GSR	0.743549	9.59*E* − 52
PRNP	0.613288	3.58*E* − 33
PRDX1	0.609423	1.24*E* − 39
HMOX2	0.551601	1.50*E* − 15
GCLC	0.545956	8.41*E* − 12
PRDX6	0.418619	3.87*E* − 18
SCAF4	0.396722	6.68*E* − 10
GLRX	0.39422	2.50*E* − 05
ATOX1	0.370471	0.001248
GLRX2	0.343887	8.97*E* − 06
GPX3	0.324067	4.18*E* − 07
SBNO2	0.095644	0.149361
ERCC2	0.071641	0.329135
TXNRD2	0.053783	0.564995
NDUFA6	0.045766	0.533123

## Data Availability

The data used to support the findings of this study are available from the corresponding author upon reasonable request.

## Abbreviations

CS: Cigarette smoke
COPD: Chronic obstructive pulmonary disease
SASP: Senescence-associated secretory phenotype
Hsp27: Heat shock protein 27
G6PD: Glucose-6-phosphate dehydrogenase
CKD: Chronic kidney disease
ROS: Reactive oxygen species
DAPI: 4′,6-Diamidino-2-phenylindole
GSEA: Gene set enrichment analysis
SA*β*gal: Senescence-associated *β*-galactosidase
IHC: Immunohistochemistry.
